# Cocoa Flavanols Adjuvant to an Oral Nutritional Supplement Acutely Enhances Nutritive Flow in Skeletal Muscle without Altering Leg Glucose Uptake Kinetics in Older Adults

**DOI:** 10.3390/nu13051646

**Published:** 2021-05-13

**Authors:** Tanvir S Sian, Ushnah S. U. Din, Colleen S. Deane, Ken Smith, Amanda Gates, Jonathan N. Lund, John P. Williams, Ricardo Rueda, Suzette L. Pereira, Bethan E. Phillips, Philip J. Atherton

**Affiliations:** 1 MRC-Versus Arthritis Centre for Musculoskeletal Ageing Research and National Institute for Health Research Nottingham Biomedical Research Centre, School of Medicine, University of Nottingham, Derby DE22 3DT, UK; tanvirsian@gmail.com (T.S.S.); ushnah.din@nottingham.ac.uk (U.S.U.D.); ken.smith@nottingham.ac.uk (K.S.); amanda.gates@nottingham.ac.uk (A.G.); jon.lund@nottingham.ac.uk (J.N.L.); john.williams7@nottingham.ac.uk (J.P.W.); 2 Department of Surgery and Anaesthetics, Royal Derby Hospital, Derby DE22 3NE, UK; 3 Department of Sport and Health Sciences, College of Life and Environmental Sciences, University of Exeter, Exeter EX1 2LU, UK; c.s.deane@exeter.ac.uk; 4 Living Systems Institute, University of Exeter, Stocker Road, Exeter EX4 4QD, UK; 5 Research and Development, Abbott Nutrition, 18004 Granada, Spain; ricardo.rueda@abbott.com; 6 Research and Development, Abbott Nutrition, Columbus, OH 43219, USA; suzette.pereira@abbott.com

**Keywords:** cocoa flavanols, blood flow, glucose metabolism, skeletal muscle, ageing

## Abstract

Ageing is associated with postprandial muscle vascular and metabolic dysfunction, suggesting vascular modifying interventions may be of benefit. Reflecting this, we investigated the impact of acute cocoa flavanol (450–500 mg) intake (versus placebo control) on vascular (via ultrasound) and glucose/insulin metabolic responses (via arterialised/venous blood samples and ELISA) to an oral nutritional supplement (ONS) in twelve healthy older adults (50% male, 72 ± 4 years), in a crossover design study. The cocoa condition displayed significant increases in *m. vastus lateralis* microvascular blood volume (MBV) in response to feeding at 180 and 240-min after ONS consumption (baseline: 1.00 vs. 180 min: 1.09 ± 0.03, *p* = 0.05; 240 min: 1.13 ± 0.04, *p* = 0.002), with MBV at these timepoints significantly higher than in the control condition (*p* < 0.05). In addition, there was a trend (*p* = 0.058) for MBV in *m. tibialis anterior* to increase in response to ONS in the cocoa condition only. Leg blood flow and vascular conductance increased, and vascular resistance decreased in response to ONS (*p* < 0.05), but these responses were not different between conditions (*p* > 0.05). Similarly, glucose uptake and insulin increased in response to ONS (*p* < 0.05) comparably between conditions (*p* > 0.05). Thus, acute cocoa flavanol supplementation can potentiate oral feeding-induced increases in MBV in older adults, but this improvement does not relay to muscle glucose uptake.

## 1. Introduction

Skeletal muscle is the largest organ system by mass in the body, and in being its locomotory engine, largest amino acid reservoir, and main site of postprandial glucose handling [1], represents an organ crucial to the maintenance of physical functioning and metabolic health. Of the conditions associated with skeletal muscle deterioration, age-related sarcopenia represents a global health problem, demonstrating robust associations with risk of frailty [2], morbidity [3], and in extreme cases, mortality [4]. Ageing is also the main risk factor for cardiovascular disease [5], with deterioration of vascular systems feeding directly into skeletal muscle implicated in sarcopenia [6]. Reflecting this, older adults display reductions of ~20–30% in limb conduit artery blood flow compared to younger counterparts [7,8], underpinned by reductions in capillary density and surface area [9,10]. Additionally, reduced muscle microvascular blood flow is observed in response to anabolic stimuli (e.g., feeding) [9,11], with nutrient-induced increases in whole-limb perfusion being attenuated with age [8]. Such blunted micro- and macrovascular blood flow responses are hypothesised to contribute to age-related “anabolic resistance” to feeding, via the limited delivery and/or utility of insulin and amino acids by muscle [12]. Since vascular dysfunction appears to be a modifiable, rather than inevitable aspect of chronological ageing [11] (as evidenced by the preservation and improvement in vascular function in lifelong exercisers and those starting exercise training, respectively [13,14]), determining therapeutic strategies for maintaining or even potentiating vascular responses in older adults is of paramount importance for the maintenance of muscle mass and reducing cardiovascular risk.

Flavanols, a sub-group of plant-derived phytochemicals found naturally occurring in (but not limited to) chocolate and red wine, have gained increasing attention as a potentially efficacious nutraceutical since it was shown that high intakes of cocoa were associated with a lower incidence of vascular-related diseases [15]. Subsequently, numerous clinical trials have shown that flavanol-rich cocoa improves vascular endothelial (dys)function in both healthy cohorts [16] and in those with disease [17], purportedly due to, or at least in part, increased nitric oxide synthase activity and thus bioactive nitric oxide [18]. To demonstrate, cocoa flavanol-induced vasodilation in humans was reversed in the presence of the nitric oxide synthase inhibitor, L-NAME [19]. Further confirming these findings, cocoa flavanols increased circulating nitric oxide species (RXNO) in tandem with enhanced vasodilation, which were both reversed by L-NAME [20]. In line with this, we have previously shown that acute cocoa supplementation improved muscle vascular responsiveness to intravenous nutrition in older men [21]. However, these results may not represent the effects of cocoa when combined with oral feeding where additional factors such as gut hormones (e.g., incretins) may have an effect [21]. Indeed, demonstrating the potency of gut hormones on these responses, the incretin hormone, glucagon-like peptide-1, has been shown to markedly enhance postprandial microvascular perfusion [22]. As such, it is plausible that cocoa supplementation to oral feeding may modulate vascular responses in older adults, though this remains to be determined.

Importantly, enhanced vascular responses may translate to improved glucose metabolism. This may occur via the greater insulin-mediated glucose delivery to, and utilisation by, skeletal muscle which is typically attenuated as part of the insulin resistance that develops with advancing age and has been shown to contribute to the development of sarcopenia [23]. Indeed, impaired glucose handling can be modified by nutraceutical intervention in the presence of improved endothelial function [24]. In the context of cocoa flavanols, chronic consumption has been shown to improve the metabolic profile of older adults via reductions in insulin resistance [25], which may lead to increased glucose uptake as directly evidenced in pre-clinical models using skeletal muscle cells [26]. Nonetheless, whether vascular responses subsequently impact insulin kinetics and glucose uptake following acute cocoa flavanol intake in healthy older adults remains poorly defined.

It is evident that even ostensibly healthy older adults exhibit reductions in aspects of limb and muscle vascular function alongside impaired glucose handling. Therefore, the aim of this study was to assess the impact of acute cocoa flavanol supplementation on oral feeding-induced changes in; (i) macrovascular (limb) blood flow, (ii) muscle microvascular blood flow, and (iii) metabolic responses, in healthy older adults.

## 2. Materials and Methods

### 2.1. Ethical Approval

This study was reviewed and approved by the University of Nottingham Faculty of Medicine and Health Sciences Research Ethics Committee (2-1704) and was conducted in accordance with the Declaration of Helsinki. All study associated risks and procedures were thoroughly explained to volunteers and written consent was obtained prior to participation. This study was pre-registered at clinicaltrials.gov (NCT03213340).

### 2.2. Volunteers and Study Design

Healthy older adults (≥65 years) were recruited from the local community and/or via an internal recruitment database for this randomised, single-blind, placebo-controlled, crossover clinical trial. Volunteers were eligible for the study if they met all of the following inclusion criteria, determined at an initial screening session: (i) ≥65 years of age; (ii) body mass index between 18–30 kg/m^2^; (iii) free from active metabolic disease assessed by past medical history and had a clinically normal blood profile (liver and kidney function; complete blood count; HbA1c <6%); (iv) blood pressure <160/100 mmHg; and (v) able to provide written informed consent. Volunteers were excluded if they: were unable to adhere to the study protocol; performed regular formal exercise (or any other routine strenuous physical activity) more than once a week; were smokers; had surgery within past 3 months; had cerebrovascular disease or active cardiovascular, respiratory, inflammatory bowel or renal disease; were taking beta-adrenergic blocking agents; had active malignancy or until confirmed remission; had clotting dysfunction; had a history of deep vein thrombosis; had significant musculoskeletal or neurological disorders; had a family history of early (<55 years) death from cardiovascular disease; had known sensitivity to Sonovue™ contrast agent; had known allergy or intolerance to any of the study ingredients; and/or were regularly taking over the counter supplements containing cocoa flavanols. Volunteers also completed a handgrip strength assessment and short physical performance battery during this session.

**Figure 1:**
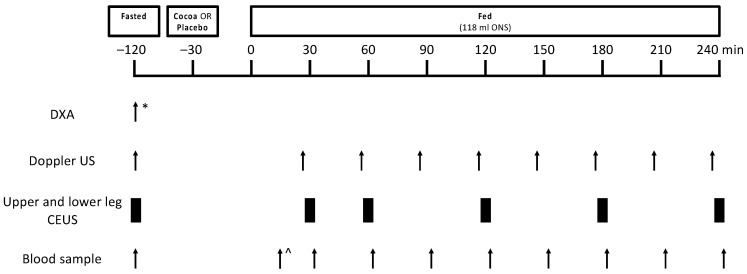
Schematic representation of the study protocol for experimental study visits 1 and 2. Twelve healthy older adults were studied in a crossover design in the fasted state, and with and without cocoa flavanols in the fed state (via oral nutritional supplement). * indicates assessment was carried out during study visit 1 only, ^ indicates that the first blood draw occurred 15 min after mixed meal feeding. CEUS, contrast-enhanced ultrasound; DXA, dual-energy X-ray absorptiometry; ONS, oral nutritional supplement; US, ultrasound.

After the screening session, for those volunteers who were deemed eligible, this study included two experimental study visits, separated by a 10–15 day “wash-out” period (Figure 1). Volunteers were instructed to refrain from heavy exercise for 48 h prior to each study visit and from taking medications that may impact blood flow on the day prior to and on the day of each study visit (e.g., angiotensin-converting enzyme inhibitors, decongestants). On the day of testing, volunteers arrived fasted from the night before (water ad libitum) and had lean leg mass measured via dual X-ray absorptiometry (DXA; Luna Prodigy II; GE Medical Systems, Little Chalfont, Buckinghamshire, UK) (study visit 1 only), leg blood flow (LBF) measured via Doppler ultrasound (iU22; Philips Healthcare, Reigate, UK), microvascular blood flow (MBF) measured via contrast-enhanced ultrasound (CEUS; iU22) and a baseline blood sample taken. Volunteers were then provided with the study supplement (cocoa or placebo) to consume orally. An oral nutritional supplement (ONS) was consumed 30 min after the study supplement. Thereafter, Doppler ultrasound measurements, CEUS measurements and blood samples were obtained periodically over the subsequent ~4 h period. Volunteer characteristics are shown in Table 1.

### 2.3. Study Supplements and ONS Feeding

Volunteers were randomly assigned to receive either the cocoa or placebo supplement in a crossover design. The placebo supplement was 33 g of low flavanol white chocolate chips (Blommer Royal, East Greenville, PA, USA) providing <50 mg flavanols (control). The cocoa supplement was 33 g of high flavanol cocoa chips (Acticoa, Barry Callebaut, Lebbeke-Wieze, Belgium) proving 450–500 mg cocoa flavanols (cocoa). The 450–500 mg dose was chosen as it is similar to the acute dose previously shown to elicit favourable changes in cardiovascular function indices [27,28] and blood flow responses [21]. Moreover, the chosen dose closely represents the dose given chronically, which has been shown to improve endothelial function [29]. Supplements were isocaloric and matched for taste and appearance. Exactly 30 min after consuming the supplement, volunteers consumed 118 mL of ONS (Ensure Advance Vanilla, Abbott, North Chicago, IL, USA) providing 175 kcal, 7.5 g protein, 24 g carbohydrate and 6 g fat, in order to investigate whether cocoa flavanols can enhance vascular responses beyond those achieved with mixed macronutrient oral feeding.

### 2.4. Measurement of LBF Using Doppler Ultrasound

LBF was measured by Doppler ultrasound (iU22 ultrasound scanner, Phillips Healthcare, Reigate, Surrey, UK), as previously described [30]. In brief, a L17-5 MHz probe was positioned over the left common femoral artery to facilitate the assessment of LBF as vessel cross-sectional area x mean velocity, over 6 cardiac cycles. Ultrasound gel was used to enhance the ultrasound signal, with all measurements taken with the volunteer supine with no visual or aural stimuli. A mean of three measurements was made at each timepoint, distributed across the study period. LBF was adjusted to lean leg mass for each volunteer and standardised to fasting LBF. Leg vascular conductance (LVC) was calculated as: LBF/mean arterial pressure (which was calculated as: (2/3 diastolic blood pressure) + (1/3 systolic blood pressure)) and leg vascular resistance (LVR) was calculated as: mean arterial pressure/LBF, as described previously [12,31].

### 2.5. Measurement of MBF Using CEUS

CEUS permitted the measurement of changes in MBF and its components: microvascular blood volume (MBV) and microvascular flow velocity (MFV). As previously described in detail [32], a iU22 ultrasound scanner (Phillips Healthcare, Reigate, Surrey, UK) was used to detect Sonovue™ microbubbles (Bracco, Milan, Italy), which were infused via an antecubital fossa vein. Briefly, one linear probe was positioned on the *m. vastus lateralis* and another on the *m. tibialis anterior* to detected intravascular microbubble concentration in both upper and lower leg muscles. Intermittent high mechanical index “flashes” were used to disrupt microbubbles, with subsequent continuous low mechanical index recording measuring the rate of microbubble reappearance after each flash. Sonovue™ was first infused at 2 mL/min for 1 min and then 1 mL/min for 3 min thereafter. At 2.5 min 3–30 s flash/replenishment recordings were made across the last 90 s of this protocol at each CEUS timepoint. After each flash, a 0.48 s window was used to adjust for non-contrast signal and for rapid filling of larger conduit (non-exchange) vessels. The acoustic intensity of the insonated tissue in the post-flash period demonstrates a first order exponential association function with a rate constant that is proportional to MFV and a plateau proportional to MBV. For each CEUS measurement volunteers were asked to rest still and quiet, with each measurement lasting <10 min.

### 2.6. Blood Sampling

Glucose uptake/release was assessed using an Arterio-Venous (A-V) sampling approach, by measuring blood glucose concentrations (Glucose Analyzer, YSI, Yellow Springs) across the leg by sampling arterial and venous bloods (using the Fick Principle) [33]. Plasma insulin concentrations was measured using a high sensitivity human insulin enzyme-linked immunosorbent assay (ELISA; DRG Instruments GmbH, Marburg, Germany) according to manufacturer’s instruction. Total insulin responses to feeding for each volunteer was calculated using the area under the insulin concentration/time curve above baseline (with baseline equal to insulin concentration measured before feeding).

### 2.7. Statistical Analysis

Two-way repeated measures ANOVA with Dunnett’s multiple comparison analysis was used to determine time x supplement effects. CEUS data was normalised to baseline to allow comparison between conditions. Two-way repeated measures ANOVA with Sidak’s post hoc analysis was used to compare condition differences. Blood glucose A-V balance and insulin data were analysed by area under the curve (AUC) with paired t-tests used to determine supplement effects. Data were accepted as significant if *p* < 0.05. Trends were reported and interpreted with caution if *p* < 0.10. Data analysis was conducted using GraphPad Prism version 8 (GraphPad Software, San Diego, CA). Data are presented as mean ± SEM (unless otherwise stated).

## 3. Results

### 3.1. LBF, LVC and LVR

**Figure 2:**
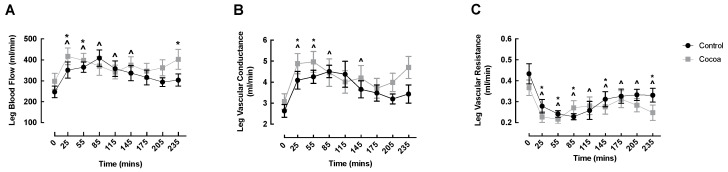
Cocoa did not enhance oral nutritional supplement feeding-induced changes in leg blood flow (**A**), vascular conductance (**B**) or vascular resistance (**C**) of healthy older adults. ^ denotes significant within condition difference from control baseline (*p* < 0.05); * denotes significant within condition difference from cocoa baseline (*p* < 0.05).

LBF significantly increased from baseline in both conditions early in the fed phase (cocoa: 0 min: 297.70 ± 38.17 vs. 25 min: 416.89 ± 40.08 mL/min, *p* = 0.001; control: 0 min: 247.19 ± 27.77 vs. 25 min: 351.23 ± 38.53 mL/min, *p* = 0.007), returning to basal levels by 85 min in the cocoa condition, and by 175 min in the control condition (Figure 2A). Interestingly, compared to baseline there was a second increase in LBF in the cocoa condition at 235 min (0 min: 297.70 ± 38.17 vs. 235 min: 402.58 ± 47.52 mL/min, *p* = 0.007) (Figure 2A). Similar to LBF, LVC significantly increased from baseline in both conditions early in the fed phase (cocoa: 0 min: 3.08 ± 0.37 vs. 25 min: 4.88 ± 0.48 mL/min, *p* = 0.007; control: 0 min: 2.63 ± 0.30 vs. 25 min: 4.09 ± 0.42 mL/min, *p* = 0.007), again returning to basal values by 85 min in the cocoa condition and by 175 min in the control condition (Figure 2B). LVR was significantly decreased at all timepoints in both conditions, except at 115, 175 and 205 min in the cocoa condition (Figure 2C). There were no significant differences between conditions at any timepoint for any Doppler-based measurements.

### 3.2. MBF

**Figure 3:**
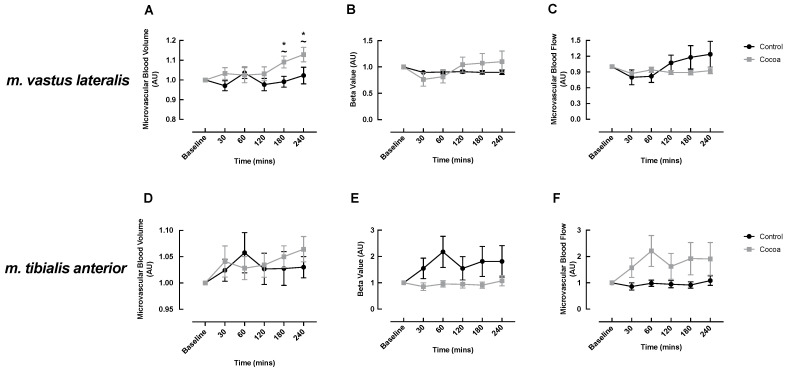
Cocoa enhanced oral nutritional supplement feeding-induced changes in microvascular blood volume (**A**) but not microvascular flow velocity (**B**) or microvascular blood flow (**C**) in the *m. vastus lateralis* and did not influence microvascular perfusion of the *m. tibialis anterior* (**D**–**F**) of healthy older adults. ~ denotes a significant difference between conditions (*p* < 0.05); * denotes significant within condition difference from cocoa baseline (*p* < 0.05).

CEUS measurements were performed on the *m. vastus lateralis* and *m. tibialis anterior* of healthy older adults to determine the effects of acute cocoa flavanol supplementation on MBV, MFV and their product MBF in muscles that differ in both location (proximal vs. distal, respectively), size (larger vs. smaller, respectively) and fibre type composition (equal type I:II ratio vs. predominantly type I, respectively). In the *m. vastus lateralis*, the cocoa condition demonstrated a significant increase in MBV responses to an ONS at 180 and 240 min post-feeding compared to baseline (baseline: 1.00 vs. 180 min: 1.09 ± 0.03, *p* = 0.05; 240 min: 1.13 ± 0.04, *p* = 0.002) with MBV at these timepoints significantly higher than those observed in the control condition (180 min: cocoa: 1.09 ± 0.03 vs. control: 0.99 ± 0.03, *p* = 0.033; 240 min: cocoa: 1.13 ± 0.04 vs. control: 1.02 ± 0.04, *p* = 0.021) (Figure 3A). Interestingly, the increase in MBV at 240 min closely coincided with the secondary increase in LBF seen in the cocoa condition (Figure 2A). In the control condition, there was no significant change in MBV from baseline over time. MFV and MBF did not change over time within the cocoa or control condition and no significant differences were detected between the conditions (Figure 3B,C).

In the *m. tibialis anterior*, MBV, MFV and MBF did not change over time within the cocoa or control condition and there were no significant differences between the conditions (Figure 3D–F). However, there was a trend for increased MBV in the cocoa condition at 240 min post-meal (baseline: 1.00 vs. 240 min: 1.06 ± 0.03, *p* = 0.058), which was not observed in the control condition (Figure 3D).

### 3.3. Blood Glucose and Insulin

**Figure 4:**
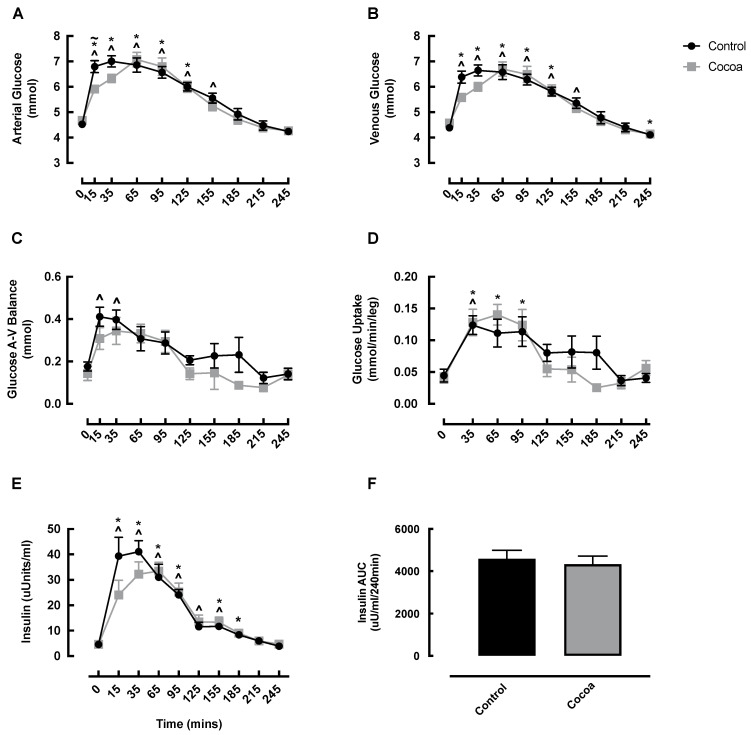
Changes in arterial glucose (**A**), venous glucose (**B**), glucose A-V balance (**C**), glucose uptake (**D**), insulin (**E**) and insulin AUC (**F**) in healthy older adults in response to cocoa or control, following oral nutritional supplement feeding. ~ denotes a significant difference between conditions (*p* < 0.05); ^ denotes significant difference from control baseline (*p* < 0.05); * denotes significant difference from cocoa baseline (*p* < 0.05). AUC, area under the curve; A-V, arterio-venous.

Arterial and venous glucose both significantly increased in the early post-feeding phase in both the cocoa and control condition, which was expedited compared to the control condition, and returned to baseline by 185 min (Figure 4A,B). No significant difference was observed between the conditions at any timepoint except for arterial glucose at 15 min, which was higher in the control condition (Figure 4A). Arterial and venous AUC was not different between conditions (data not shown). Glucose A-V balance increased at 15 (0 min: 0.18 ± 0.02 vs. 15 min: 0.41 ± 0.05 mmol, *p* = 0.009) and 35 min (0 min: 0.18 ± 0.02 vs. 35 min: 0.40 ± 0.05 mmol, *p* = 0.026) in the control conditions only, however, there was no significant differences between conditions for glucose A-V balance at any timepoint (Figure 4C), or for glucose AUC (data not shown). Glucose uptake increased in both conditions at 35 min (cocoa: 0 min: 0.04 ± 0.01 vs. 35 min: 0.13 ± 0.02 mmol/min/leg, *p* = 0.021; control: 0 min: 0.04 ± 0.01 vs. 35 min: 0.12 ± 0.01 mmol/min/leg, *p* = 0.012) and remained elevated only in the cocoa condition at 65 and 95 min (0 min: 0.04 ± 0.01 vs. 65 min: 0.14 ± 0.02 mmol/min/leg, *p* = 0.011; vs. 95 min: 0.12 ± 0.02 mmol/min/leg, *p* = 0.041), however, there was no significant difference between conditions at any time point for glucose uptake (Figure 4D) or glucose uptake AUC (data not shown). As expected, insulin significantly increased from baseline both conditions early post-feeding, returning to basal values by 185 min in the control condition and 215 min in the cocoa condition (Figure 4E). There was no significant difference between the conditions at any timepoint in insulin, or for insulin AUC (Figure 4E,F).

## 4. Discussion

We investigated if acute cocoa flavanol supplementation coupled to oral feeding would modify micro- and macrovascular responses in the leg, and subsequent metabolic responses, in healthy older adults. We found that acute supplementation of cocoa flavanols increased micro- but not macrovascular responses to ONS; these microvascular changes did not translate into improved glucose/insulin responses.

Our primary finding was that acute cocoa flavanol supplementation may overcome age-related vascular dysfunction in the postprandial state (in the context of the *m. vastus lateralis*), suggesting that the impact of cocoa flavanols goes beyond the effect of the feeding-related insulin response, which alone is known to enhance microvascular flow [34]. As enhanced MBV is observed following acute cocoa supplementation, and since we have found plasma epicatechin to peak 2 h following cocoa flavanol consumption (baseline: none detected, 2 h: 0.21 ± 0.06 µM, undetectable following placebo), it is likely that increased bioactive nitric oxide availability underlie these changes [19]. Interestingly, increases in MBV were only observed later in the postprandial period (180 and 240 min), yet in a previous study, we observed that net essential amino acid (EAA) uptake had returned to fasting levels by 180–240 min [35]. It is possible that the observed changes in MBV reflect late capillary recruitment and may not impact EAA-induced increases in muscle protein synthesis. This notion is supported by our previous work which found acute cocoa flavanols increased MBV in the muscles of older men when administered with an intravenous infusion of amino acids, but this did not translate into augmented protein accretion [21]. As such, the physiological impacts of enhanced acute vascular responses, as seen following cocoa supplementation, remain to be determined. It is plausible that these vascular responses can lead to increased nutrient (and oxygen) delivery to the muscle, laying the foundations for longer-term adaptations with chronic supplementation. In support of this, chronic flavanol supplementation has been shown to (i) improve exercise capacity, accompanied by enhanced oxidative capacity and angiogenesis (albeit in a pre-clinical model) [36]; (ii) improve mitochondrial structure and biogenesis in type II diabetic and heart failure patients [37]; and (iii) improve capillary density in individuals with peripheral artery disease [38]. Therefore, we anticipate that cocoa supplementation has important physiological consequences beyond acute anabolism, which require further investigation in the form of chronic supplementation human trials.

To our knowledge, this is the first trial to assess the impacts of acute cocoa flavanol supplementation in both the *m. vastus lateralis* and the *m. tibialis anterior*. Whilst previous studies in humans have started to unravel cocoa flavanol-induced impacts on muscle perfusion, to date they have mainly focused on the *m. vastus lateralis*; a proximal muscle that displays a roughly equal fibre type split [39]. Currently, little is known about more distal, oxidative, capillary-dense muscles such as *m. tibialis anterior* [40], which is composed of ~70% type I fibres, irrespective of gender [41]. Considering the greater capillarisation of the *m. tibialis anterior*, it is plausible that cocoa flavanols may be beneficial in such muscles due to the potential for greater capillary recruitment and thus greater delivery of insulin and amino acids. Functionally, the *m. tibialis anterior* is the primary ankle dorsiflexor underpinning everyday activities, such as gait and balance when walking [41], which are increasingly important during ageing and strongly associate with the occurrence of falls [42]. Therefore, the ability to improve the vascular profile of lower limb muscles, alongside upper limb muscles, could have significant ramifications for whole limb functionality/muscle health in ageing populations. Considering that we found a trend (*p* = 0.06) towards increased MBV at 240 min in the cocoa condition only is suggestive that cocoa *may* also increase late capillary recruitment within the *m. tibialis anterior*. That these effects appear to be attenuated in comparison to those seen in the *m. vastus lateralis* may be due to its smaller size and thus absolute vascular network size, rendering potentially impactful changes smaller, and therefore harder to detect, compared to the larger *m. vastus lateralis*.

As well as MBV, CEUS also permits the measurement of other vascular components, namely microvascular blood flow velocity (i.e., MFV) and the combined product of MBV and MFV that is MBF [11]. We found that neither ONS or ONS plus cocoa flavanols had any impact on MFV, and despite changes in MBV, MBF did not change in either *m. vastus lateralis* or *m. tibialis anterior*. Seeing as MFV is dependent on the dilation of the resistance arterioles that are upstream of the terminal arterioles and capillaries, it is plausible that longer term supplementation protocols are needed to reveal the effect of cocoa flavanols on MFV and MBF. In addition, it should be noted that it is MBV that has been shown, since the classical studies of August Krogh, to be associated with tissue metabolism [43,44] and confirmed to represent capillary perfusion [44,45].

Despite cocoa-specific impacts on MBV, LBF (i.e., macrovascular blood flow) increased similarly in the cocoa and control conditions, indicating that LBF is primarily driven by the insulin response to ONS and cannot be further potentiated by acute cocoa flavanol intake. However, it is not wholly surprising that MBV does not change proportionally to LBF as the two have been previously shown not to correlate [34], and changes in MBV can occur with little change in LBF [46]. Indeed, the insulin-mediated (i.e., via the ONS) decrease in LVR and increase in LVC observed in both the cocoa and control conditions are the probable primary drivers behind augmented LBF [47]. Although somewhat speculative, we observed an identical temporal response in LBF and LVC, which may suggest that LVC is the critical determinant of the LBF response (compared with LVR), however, this requires further validation. Further, although ONS stimulated beneficial responses in LVC and LVR, these were not potentiated by acute cocoa flavanols, suggesting chronic cocoa supplementation is required to drive LBF, LVC and LVR adaptations. In support of this, numerous chronic studies in the literature have reported beneficial macrovascular outcomes in young [48] and middle-aged [28] healthy adults after cocoa supplementation, with chronic investigation in healthy older adults still required.

We also determined whether cocoa-induced improvements in vascular responses translated into improved insulin/glucose metabolic responses; a hypothesis worthy of investigation since chronic intake of high dose cocoa flavanols can improve metabolic outcomes in type II diabetics [49]. Despite a valid rationale and the observed increase in MBV, although plasma insulin increased following ONS, there were no temporal differences between the cocoa and control condition. This suggests that a further increase in insulin is not required for potentiated MBV responses, supporting our previous work which also found no differences in plasma insulin or proximal insulin signalling following acute cocoa supplementation [21]. Similarly, the increase in glucose uptake following ONS was similar between conditions and therefore not potentiated by cocoa flavanols. Seeing as muscle glucose uptake is driven by insulin, the similar insulin responses between conditions may explain comparable glucose uptake levels. As insulin and glucose kinetics were similar between the cocoa and control conditions in this acute study, it is likely that it is the cumulative effect of cocoa flavanols (i.e., chronic supplementation) that elicits the previously observed favourable metabolic responses (e.g., [49]), and thus this form of supplementation regime requires testing in older healthy (i.e., non-diabetic) adults, who as a consequence of advancing age are at heightened risk of cardiometabolic disease [50].

Regarding potential study limitations, although this study recruited older adults to investigate the impact of cocoa flavanols on aspects related to age-related comorbidities, we acknowledge that by using *healthy* older adults our volunteer pool may not be truly representative of the older adult population as a collective, given the known prevalence of comorbidities and associated polypharmacy in older age [51]. Additionally, our study sample (*n* = 12) may be considered small and so larger clinical trials are needed to confirm the vascular-promoting effects of cocoa flavanols in larger cohorts. Nonetheless, our study is strengthened by the crossover design, which allows volunteers to serve as their own control therein minimising the influence of confounding factors (e.g., body mass), thus requiring smaller volunteer numbers to achieve sufficient power compared to parallel study designs [52,53]. Further, post hoc power calculation using the current dataset output power as >0.8, suggesting our sample size was sufficient to detect statistical significance. Furthermore, studies investigating blood flow responses in young [54] and older [7] adults have used fewer volunteers than herein (i.e., *n* = 6–8).

## 5. Conclusions

To conclude, we show that acute cocoa flavanol supplementation can potentiate ONS feeding-induced increases in MBV in healthy older adults, but this improvement does not translate to enhanced glucose/insulin metabolism. The acute cocoa flavanols provided during this study were consumed orally and were well-tolerated by all volunteers, suggesting that cocoa flavanols may be an efficacious and safe nutraceutical intervention for increasing muscle blood flow, and thus nutrient and oxygen delivery, in older adults. Larger clinical trials investigating the effects of acute and chronic cocoa supplementation should be conducted to confirm these findings, and the potential physiological impact, in older adults before recommending cocoa flavanols as vascular promoting nutraceuticals.

## Figures and Tables

**Table 1:** Volunteer characteristics (mean ± SD).

Parameter	Volunteers (*n* = 12)
Gender (% M)	50
Age (years)	72 ± 4
Height (cm)	170.7 ± 6.8
Weight (kg)	74.0 ± 13.1
BMI (kg/m^2^)	25.3 ± 3.1
Lean mass (kg)	46.5 ± 8.4
Resting heart rate (bpm)	65 ± 8
Resting systolic blood pressure (mmHg)	129 ± 10
Resting diastolic blood pressure (mmHg)	78 ± 8
Grip strength (kg) *	30.14 ± 7.79
SPPB	10 ± 1.1

BMI, body mass index; SPPB, short physical performance battery. * *n* = 11.

## Data Availability

The data presented in this study are available on request from the corresponding authors.
